# Multi-site, *in vivo* MRI dataset of brain diffusivity measures before and after harmonization, and atrophy measures following controlled cortical impact in male and female adult rats

**DOI:** 10.3389/fneur.2025.1719618

**Published:** 2025-12-15

**Authors:** Gregory Kislik, Rachel Fox, Alexandru Korotcov, Jinyuan Zhou, Marcelo Febo, Babak Moghadas, Adnan Bibic, Yunfan Zou, Jieru Wan, Raymond C. Koehler, Temitope Adebayo, Mark P. Burns, Joseph T. McCabe, Kevin K. W. Wang, J. Russell Huie, Adam R. Ferguson, Afshin Paydar, Ina B. Wanner, Neil G. Harris, The TOP-NT Investigators

**Affiliations:** 1 Department of Neurosurgery, University of California at Los Angeles Brain Injury Research Center, David Geffen School of Medicine, Los Angeles, CA, United States; 2 Department of Radiology and Bioengineering, Uniformed Services University of the Health Sciences, Bethesda, MD, United States; 3 Henry M. Jackson Foundation for the Advancement of Military Medicine, Bethesda, MD, United States; 4 Department of Radiology, Johns Hopkins University, Baltimore, MD, United States; 5 Department of Psychiatry, University of Florida, Gainesville, FL, United States; 6 Hugo W. Moser Research Institute at Kennedy Krieger, Baltimore, MD, United States; 7 Department of Anesthesiology and Critical Care Medicine, Johns Hopkins University, Baltimore, MD, United States; 8 Department of Neuroscience, Georgetown University Medical Center, Washington, DC, United States; 9 Department of Anatomy, Physiology and Genetics, Uniformed Services University of the Health Sciences, Bethesda, MD, United States; 10 Department of Neurobiology, Morehouse School of Medicine, Atlanta, GA, United States; 11 Department of Neurological Surgery, University of California, San Francisco, CA, United States; 12 Semel Institute for Neuroscience and Human Behavior, University of California at Los Angeles, Los Angeles, CA, United States; 13 Intellectual Development and Disabilities Research Center, University of California at Los Angeles, Los Angeles, CA, United States

**Keywords:** traumatic brain injury, controlled cortical impact, diffusion MRI, fractional anisotropy, multi-site harmonization

## Abstract

Traumatic brain injury (TBI) research faces persistent challenges in data comparability and reproducibility, particularly in multi-center preclinical studies. Structured, interoperable datasets are essential to identify robust imaging biomarkers and validate cross-site findings. This dataset comprises 343 diffusion-weighted MRI scans from 186 male and female Sprague Dawley rats subjected to controlled cortical impact (CCI) or sham procedures at four independent research sites. Imaging was performed at 3 and 30 days post-injury using harmonized acquisition protocols and field strengths ranging from 7T to 11.7T. A standardized processing pipeline generated scalar diffusion maps (FA, MD, AD, and RD), anatomical templates, and voxel-wise *z*-score–based indices of injury. Both unharmonized and harmonized versions of the dataset are provided. Harmonization was performed using NeuroCombat for scalar volumes and multi-site template registration for voxel-level alignment. The dataset supports investigations into sex, post-injury timepoint, and injury effects in experimental TBI and provides a foundation for testing image harmonization methods, developing quantification tools for automated assessment of injury, and training machine learning models. The dataset is published in the FAIR² framework, with machine-actionable metadata, responsible AI indicators, and structured documentation to support ethical, reproducible, and AI-ready reuse.

## 1 Introduction

In the pursuit of advancing reproducible and translatable models of traumatic brain injury (TBI), it is essential to generate and harmonize preclinical datasets that reflect the variability of biological responses while minimizing technical confounds. The dataset presented here, developed by the Translational Outcomes Project in NeuroTrauma (TOP-NT) Consortium (3), addresses a persistent limitation in the field: the lack of multi-site, harmonized diffusion MRI data that enables robust analysis of injury effects across experimental sites, imaging platforms, and animal cohorts. The motivation for creating this dataset arises from the need to quantify structural brain changes following controlled cortical impact (CCI) using diffusion MRI metrics that are sensitive to microstructural alterations.

Preclinical TBI research has traditionally relied on single-site studies that, while well-controlled, are constrained by limited generalizability. Differences in scanner hardware, acquisition parameters, and injury protocols often result in inconsistent findings and poor reproducibility. This dataset addresses these challenges by implementing standardized injury and imaging procedures across four independent research institutions, ensuring consistency in the delivery of CCI injury and diffusion imaging while capturing natural biological variability across sexes, timepoints, and injury severities.

The dataset includes 343 diffusion-weighted imaging (DWI) scans from 186 male and female Sprague Dawley rats, acquired at two timepoints—3 and 30 days post-injury—following either a CCI or sham procedure. Imaging was conducted using Bruker systems with magnetic field strengths of 7T to 11.7T. All sites followed a harmonized acquisition protocol, and the resulting data underwent standardized preprocessing, including skull stripping, denoising, distortion correction, and tensor fitting using established neuroimaging toolkits. Scalar maps of fractional anisotropy (FA), mean diffusivity (MD), axial diffusivity (AD), and radial diffusivity (RD) were computed and registered to study-specific and multi-site templates to enable both univariate and voxel-level analysis.

To facilitate cross-site comparability, the dataset includes scalar volume estimates of abnormal tissue derived from *z*-score thresholds relative to sham distributions, both before and after harmonization. Harmonization was performed using the NeuroCombat algorithm (1) for univariate correction and template-based registration for voxel-level standardization. In addition, deformation-based morphometry was applied to assess local tissue atrophy based on Jacobian determinants.

This dataset provides a comprehensive foundation for research focused on understanding structural brain changes following TBI, validating harmonization methods, and modeling biological variables such as sex and temporal progression. Its structured format supports multi-institutional collaboration, comparative analyses, and the development of computational tools to improve TBI diagnostics and therapeutic evaluation.

## 2 Methods summary

This study presents a harmonized, multi-site diffusion MRI dataset designed to investigate structural brain alterations following controlled cortical impact (CCI) injury in rats. Data were collected across four research institutions using standardized injury protocols (2) and imaging sequences, and processed using a unified pipeline for quality control, spatial normalization, and statistical harmonization. The dataset includes univariate measures derived from scalar diffusion maps (FA, MD, AD, and RD) that were *z*-scored to produce volumetric indices of abnormal tissue, and local measures of anatomical deformation. This methods summary outlines the full workflow used to generate the dataset, structured by methodological domain.

### 2.1 Study design

A total of 186 adult Sprague Dawley rats were enrolled across UCLA, JHU, GU/USU, and UF/MSM. Subjects were balanced by sex and randomly assigned to one of two experimental conditions—CCI or sham. Imaging was conducted at two post-injury timepoints (3 and 30 days), enabling longitudinal assessment of injury effects. Each animal received a globally unique identifier to ensure traceability across imaging, processing, and analysis stages. Site participation was coordinated under shared standard operating procedures, with adaptations documented for each site's infrastructure. The study design was structured to support reproducibility, cross-site harmonization, and stratified analyses across injury, time, and sex.

### 2.2 Instrumentation and sample preparation

CCI surgery was performed under harmonized conditions using a Leica electromagnetic impactor. The surgical and injury protocols included:

- **Anesthesia and preparation**: isoflurane was used for induction (5%) and maintenance (1.5–2%), with animals placed in stereotactic frames and temperature maintained at 37 °C.
- **Craniectomy**: a midline incision and skull window (5–6 mm diameter) were made over the left hemisphere using a cooled dental drill.
- **Impact delivery**: a beveled tip (4 or 5 mm) was positioned parallel to the dura, and injury was delivered at:

° Depth: 1.2–2.8 mm
° Velocity: 3.5–6 m/s
° Dwell time: 100–240 ms

- **Post-operative care**: analgesics were administered, and recovery monitored under temperature-controlled conditions. Sham animals underwent the same protocol without the impact step.

These procedures were standardized across sites to control for inter-machine and procedural variability.

### 2.3 Data collection

Diffusion-weighted imaging (DWI) was performed using Bruker Biospec systems at different field strengths (7T at UCLA and GU/USU; 11.7T at JHU; 11.1T at UF/MSM). Despite hardware differences, acquisition protocols were harmonized across sites. All animals were scanned using:

- A 3D single-shot spin echo diffusion EPI sequence
- 42 non-colinear gradient directions per *b*-value
- *b*-values of 0, 1,000, and 3,000 s/mm ≤ (*n* = 4, 42, 42)
- A matrix of 72 × 49 × 96 within a 3D field-of-view of 18 × 12.5 × 24 mm
- Isotropic resolution of 250 μm
- Outer-volume suppression using 5–10 mm saturation slices

Scans were performed under 1%−1.5% isoflurane in medical air. Raw Bruker-format data were archived and indexed using subject identifiers to support traceability.

### 2.4 Quality assurance and validation

Preprocessing was applied uniformly across sites to remove artifacts, enhance signal quality, and standardize tensor modeling. The following sequence of steps was performed:

- **Format conversion**: raw Bruker data were converted to NIFTI using BrkRaw.
- **Brain extraction**: FSL's BET was used, refined with joint label fusion (ANTs), and manually corrected to improve brain masks.
- **Artifact correction**: FSL's eddy was used for eddy current correction; MRtrix3 tools (dwidenoise, mrdegibbs) addressed noise and Gibbs ringing.
- **Tensor fitting**: MRtrix3's dwi2tensor computed FA, MD, AD, and RD maps from preprocessed diffusion data.

This standardized approach ensured consistency and data integrity across all samples.

### 2.5 Template registration and harmonization

To enable voxel-wise comparison, all scalar maps were spatially normalized and harmonized using a two-stage strategy:

- **Site-level registration:**

° Site-specific mean deformation templates (MDTs) were created from FA data using ANTs.
° Scalar maps were registered to their site's MDT using affine and non-linear warps.

- **Multi-site harmonization:**

° A unified multi-site MDT was generated from the site-level templates.
° Subject maps were transformed into the multi-site space for cross-site alignment.
° NeuroCombat was used to perform univariate harmonization on *z*-score maps.
° Voxel-level harmonization was applied post-registration using the same *z*-score thresholds (e.g., |*z*| > 2.0 or *z* < −3.1) to ensure anatomical consistency.

This multi-level normalization allowed for site-independent analyses of injury effects.

### 2.6 Scalar map *z*-scoring and volume computation

Scalar maps were analyzed to identify regions with significantly abnormal diffusion properties. Sham animals were used to generate site- and timepoint-specific reference maps of mean and standard deviation for each scalar metric. *Z*-score maps were computed for each injured subject by normalizing scalar values against these local sham distributions. Corrected thresholds (e.g., |*z*| > 3.1) were applied to these *z*-maps to define regions of significantly low or high scalar values. These thresholds were adjusted per site and timepoint to account for multiple comparisons and control for false positives. The resulting ScalarLOW and ScalarHIGH volumes were quantified as the total number of voxels exceeding the respective thresholds for each scalar index.

### 2.7 Anatomical deformation and atrophy estimation

To assess structural deformation following injury, Jacobian determinant maps were computed from the warp fields used during spatial normalization into mean template space. These maps captured voxel-wise estimates of local expansion or contraction relative to the common template. Sham animal Jacobians were used to generate reference distributions of deformation at each site. *Z*-score maps were then created for each injured subject, and a fixed threshold (|*z*| > 3.1) was applied to identify regions of significant atrophy or swelling. The volume of tissue showing contraction (*z* < −3.1) was computed per subject and reported as the atrophy volume. This metric provided a quantitative estimate of structural tissue loss due to traumatic injury.

## 3 Data overview

### 3.1 Data summary

The dataset represents a multi-site diffusion MRI study of controlled cortical impact (CCI) injury in adult Sprague Dawley rats, designed to support cross-institutional analysis of traumatic brain injury (TBI) outcomes and harmonization techniques. It includes 343 diffusion-weighted imaging (DWI) scans collected from 186 animals (both sexes) at four independent research institutions: UCLA, JHU, GU/USU, and UF/MSM. Imaging was performed at two post-injury timepoints (3 and 30 days) following either a CCI or sham procedure. Scalar diffusion metrics—FA, MD, AD, and RD—were derived from preprocessed diffusion tensor imaging (DTI) data. Each metric was analyzed in native, site-harmonized, and voxel-harmonized template space, with abnormal high and low volumes computed based on site-specific sham-derived *z*-score thresholds. In addition, anatomical deformation was quantified using Jacobian determinant maps to estimate localized atrophy.

The dataset includes both raw and processed imaging data, with uniform preprocessing steps applied using open-source tools (BrkRaw, FSL, MRtrix3, and ANTs) and statistical harmonization performed using the NeuroCombat algorithm. All scalar maps were registered to site-specific and multi-site templates to enable inter-subject and inter-site comparability. Key derived variables include ScalarLOW and ScalarHIGH volumes for each diffusion metric, and volumetric estimates of tissue atrophy. This structure supports investigation of injury severity, recovery dynamics, sex effects, and cross-site reproducibility in preclinical TBI research.

Designed as a reproducible resource for the neuroimaging and neuroscience communities, the dataset enables evaluation of harmonization methods, validation of imaging biomarkers, and development of machine learning models for TBI assessment. It provides a high-resolution foundation for data-driven investigations into the spatial and temporal patterns of brain injury and recovery in a controlled experimental setting. The dataset is published within the FAIR² framework, with structured, machine-readable metadata, provenance tracking, and responsible AI indicators to support ethical and reproducible reuse.

### 3.2 Quantitative summary of the dataset

#### 3.2.1 Dataset composition

The dataset consists of 343 individual scan records representing 186 animals imaged across two post-injury timepoints. Each record corresponds to a unique imaging session following either CCI or sham procedures:

- **Subjects**: 186 rats (both male and female).
- **Scans**: 343 diffusion-weighted imaging (DWI) sessions (split across day 3 and day 30 post-injury).
- **Groups**:

° CCI injury group: 240 scans.
° Sham control group: 103 scans.

- **Sites**:

° UCLA: 99 scans
° GU/USU: 96 scans
° JHU: 89 scans
° UF/MSM: 59 scans

Each scan is associated with subject metadata (e.g., sex, body weight, site, timepoint), injury metadata (e.g., model type, injury severity), and a complete set of scalar diffusion measures and derived volumetric metrics.

#### 3.2.2 Data structure and formats

The dataset is provided in structured tabular format (CSV), along with Bruker-format raw imaging files and NIfTI-encoded diffusion maps generated during preprocessing. Derived variables are harmonized and annotated for univariate, voxel-level, and multi-site registration analyses.

- **Primary format**: CSV for scalar and metadata tables.
- **Imaging data**: Bruker raw files, converted NIfTI volumes.
- **Scalar outputs**: FA, MD, AD, and RD maps per scan session.
- **Derived volumes**: *Z*-scored ScalarLOW and ScalarHIGH volumes per diffusion metric

#### 3.2.3 Feature and attribute space

Each scan record includes 41 structured variables across the following categories:

- **Categorical variables**: group assignment, sex, site, injury model, timepoint.
- **Continuous variables**:

° Body weight at time of imaging.
° Volumes of abnormal diffusion by scalar metric and harmonization level (unharmonized, harmonized, voxel-harmonized) derived from corrected *z*-score maps.
° Atrophy volume derived from Jacobian maps.

- **Harmonization levels**: each scalar includes three representations—raw (unharmonized), NeuroCombat-harmonized, and voxel-level harmonized.

#### 3.2.4 Temporal and spatial coverage

- **Timepoints**: imaging conducted at 3 and 30 days post-injury.
- **No absolute dates** are recorded; all data are relative to the post-injury timeline.
- **Sites**: four institutions across the United States, each contributing to imaging and injury induction under harmonized protocols.

#### 3.2.5 Data completeness

The dataset demonstrates high internal completeness across core variables:

- **Missingness**:

° Core metadata (subject ID, site, sex, group): 0% missing.
° Preprocessed scalar volumes: >95% complete.
° Voxel-harmonized scalar volumes: ~82% complete (missing in 18% of cases due to due to either technical scanner-related problems or to occasional deaths).

- **Validation**: skull stripping and tensor fitting steps were manually corrected or reviewed where needed. All harmonization steps applied using predefined thresholds.

#### 3.2.6 Derived and annotated features

Key derived variables include:

- **ScalarLOW and ScalarHIGH volumes**: for FA, MD, AD, and RD at each harmonization level, computed using site-specific, corrected *z*-score thresholds.
- **Atrophy volume**: based on voxel-level Jacobian determinants, capturing regions of local contraction after CCI.
- **Multi-site harmonized features**: standardized across a shared anatomical template for inter-subject comparability.

#### 3.2.7 Notable statistical patterns

- **CCI vs. sham differences**: ScalarHIGH and ScalarLOW volumes were consistently larger in the CCI group across all scalar metrics, particularly at day 3 post-injury.
- **Site effects**: NeuroCombat and spatial registration procedures reduced site-specific variance in scalar volumes while preserving injury-related contrasts.
- **Atrophy**: higher local tissue contraction was observed in CCI animals, derived from Jacobian analysis of deformation fields.

### 3.3 FAIR² compliance certification

The dataset in full compliance with the FAIR² framework, which extends the Findable, Accessible, Interoperable, and Reusable (FAIR) principles by incorporating AI Readiness (AIR) and Responsible AI (RAI) standards. The dataset was extracted from a live preclinical system, transformed using procedural scripts, and published with full data provenance and metadata traceability (Table 1).

**Table 1:** The FAIR^2^ Compliance Certification presented here was generated through a Human-in-the-Loop (HITL) process combining automated FAIR^2^ system checks with author-supplied inputs. While certain metadata fields and validations (e.g., DOI registration, schema adherence, file accessibility) are verified automatically by the FAIR^2^ platform, other elements—such as domain—specific documentation quality and Responsible AI considerations-reflect expert curation by the dataset authors.

**Criteria **	**Assessment **	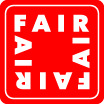
**Findability (F) **		
F1. Unique identifier	The original dataset is archived and citable via ODC-TBI DOI: https://doi.org/10.34945/F5D60C, issued by the Open Data Commons for Traumatic Brain Injury (ODC-TBI). The FAIR^2^-compliant Data Package, including harmonized data, structured metadata, and an interactive portal, is published under a distinct DOI: https://doi.org/10.71728/senscience.g7x2-a9k4, issued via the FAIR^2^ infrastructure and resolvable through DataCite. These identifiers are cross-linked and independently citable, supporting both reproducibility and downstream integration.
F2. Metadata	Extensive metadata are provided, including dataset title, full list of contributors and affiliations, experimental groups, subject identifiers, imaging modalities, institutions involved, scan counts, scalar variable descriptions, and timepoints.
F3. Metadata includes data identifiers	Each metadata entry explicitly references the dataset DOI and includes subject-level identifiers linking to the data records and corresponding scans.
F4. Searchable metadata	Metadata fields are structured using standardized vocabularies, including schema.org for dataset properties, PROV-O for provenance, and CRediT for contributor roles. These controlled vocabularies enhance machine-readability and semantic searchability across platforms.
Indexed in repositories	Indexed in the FAIR^2^ Data Portal, ODC-TBI (https://odc-tbi.org/), and DataCite.
**Accessibility (A)**	
A1. Open access	The dataset is publicly accessible under the Open Data Commons Attribution License (ODC-By v1.0), permitting unrestricted reuse, redistribution, and modification with appropriate attribution.
A2. Long-term access	ODC-TBI hosts the dataset with persistent identifiers and supports institutional archiving. No explicit mirror or redundancy strategy is described beyond institutional commitments.
API access	Programmatic access is provided via the MLCommons mlcroissant API, offering machine-readable metadata and data structure aligned with FAIR^2^. Interactive visualizations are available through the FAIR^2^ Portal for exploring cohort structure, scalar metrics, and data completeness.
**Interoperability (I)**	
I1. Standardized formats	Data are shared in open, standardized formats: (a) imaging data: Bruker format (raw), NIfTI (.nii.gz) for processed volumes; (b) tabular data: CSV for scalar outputs and metadata. All formats are widely supported by neuroimaging tools and workflows.
I2. Controlled vocabularies	The metadata schema incorporates formal controlled vocabularies including schema.org for dataset structure, PROV-O for provenance, and CRediT for contributor roles. While SNOMED or NCIT are not currently used for biological terms, alignment with domain ontologies such as OBI or UBERON could further enhance semantic interoperability.
I3. Cross-platform integration	The dataset adheres to common neuroimaging practices and uses tools like FSL, ANTs, and MRtrix3. It is compatible with workflows across R, Python, and neuroinformatics platforms such as BIDS, although the data is not yet formatted to the BIDS specification.
**Reusability (R)**	
R1. Comprehensive documentation	Detailed method sections are provided, including: (1) Animal selection and surgery, (2) MRI acquisition protocols, and (3) Preprocessing, harmonization, and statistical procedures. A structured data dictionary describes all scalar volume fields, units, and derivations.
R1.1. License	Licensed under ODC-By v1.0, allowing unrestricted reuse with proper attribution.
R1.2. Detailed provenance	Each data record is linked to its acquisition site, injury model, and processing pipeline, with clear documentation of image transformation steps. Provenance is also encoded using the PROV-O ontology, linking variables directly to the methods that generated them for machine-readable traceability.
R1.3. Domain-relevant standards	The dataset is not yet BIDS-formatted but follows widely accepted neuroimaging conventions (NIfTI, CSV). The use of DTI scalar metrics (FA, MD, AD, and RD) and Jacobian-based deformation maps meets neuroimaging reporting norms.
Versioning and updates	The dataset appears as a static version. Dataset includes version metadata. Future updates will include changelogs and semantic versioning.
**AI-readiness (AIR)**	
Structured for machine learning	The dataset includes structured tabular outputs (e.g., scalar volumes, *z*-scores) suitable for supervised learning. Variables are cleanly labeled and interpretable.
Scalable	The dataset is medium-scale (343 imaging records) and suitable for GPU-accelerated workflows. NIfTI and CSV formats support integration into high-performance computing pipelines.
Training and validation sets	The structure supports data partitioning by site, group (CCI vs. sham), timepoint, or sex. These stratifications allow clear definition of training, validation, and test cohorts.
**Responsible AI (RAI)**	
Ethical standards and misuse	As a preclinical dataset, it does not involve human subjects. However, the dataset explicitly states that it should not be used to make direct clinical predictions in humans. Users are advised to cite sources and respect contextual limitations.
Biases in the dataset	Site-level differences, scanner variation, and unequal group sizes (CCI > sham) may introduce biases. Harmonization procedures (NeuroCombat, spatial templates) address technical variability but biological biases (e.g., sex, injury, or site) may remain.
Data privacy and security	No sensitive or personally identifiable data are included. All data are derived from non-human animal models and de-identified.
Fairness and non-discrimination	The dataset includes both sexes and multiple sites, enhancing diversity. However, representation is limited to a single species and injury model, constraining generalizability to other models or species.
Explainability and interpretability	Variables are documented with clear definitions and units. Input features used in machine learning are described, including transformations.
Data provenance and accountability	All derived features (e.g., *z*-scored volumes, FA/MD) are well-documented and interpretable. Scalar metrics are standard in diffusion imaging, facilitating model explainability.
Transparency and reporting	All processing steps and variable definitions are clearly documented, enabling full traceability and responsible reuse.
AI safety and fairness	Known risks include overfitting to site or injury-specific features. The inclusion of harmonized and raw volumes enables safety testing against dataset-induced model artifacts.
Ethical and social impact	All animal procedures were conducted in accordance with institutional animal care and use guidelines at each participating site, and approved by their respective Institutional Animal Care and Use Committees (IACUCs). All protocols adhered to NIH guidelines for the care and use of laboratory animals. While the dataset does not involve human subjects and should not be used to make clinical predictions, users are expected to cite sources and respect ethical constraints associated with animal research.
Human-in-the-loop (HITL) considerations	Manual corrections were applied during preprocessing (e.g., skull stripping, quality control) to correct automated outputs and ensure anatomical accuracy. These expert-driven steps introduce structured human judgment into the dataset. Users developing or evaluating HITL systems should consider that such annotations reflect preclinical domain expertise but are not formally structured as training labels. While the dataset could support HITL workflows, additional curation or annotation standards may be required depending on the application.

#### 3.3.1 Overall FAIR² badge compliance

**Compliant**—The dataset meets all core FAIR² domains: it is findable via a citable DOI and indexed metadata; accessible through open licensing, API endpoints, and visual dashboards; interoperable through standardized formats and vocabularies; reusable via structured documentation and machine-readable provenance; and AI-ready with harmonized features suitable for model training and validation. Responsible AI principles are addressed through transparent processing, ethical approval for all animal procedures, and clear limitations on reuse. Minor improvements—such as enhanced ontology alignment and API filtering capabilities—could further strengthen compliance.

## 4 Visual overview

This section highlights key patterns captured in the dataset, including the reduction of inter-site variability through harmonization, the elevation of diffusion abnormalities in injured animals, and the structural separation of groups in multivariate space. The figures illustrate how harmonized scalar metrics—fractional anisotropy, mean diffusivity, axial diffusivity, and radial diffusivity—capture acute and subacute injury effects across timepoints. These visual summaries underscore the dataset's capacity to support cross-site comparisons, biomarker discovery, and machine learning applications in preclinical neurotrauma research.

**Figure 1:**
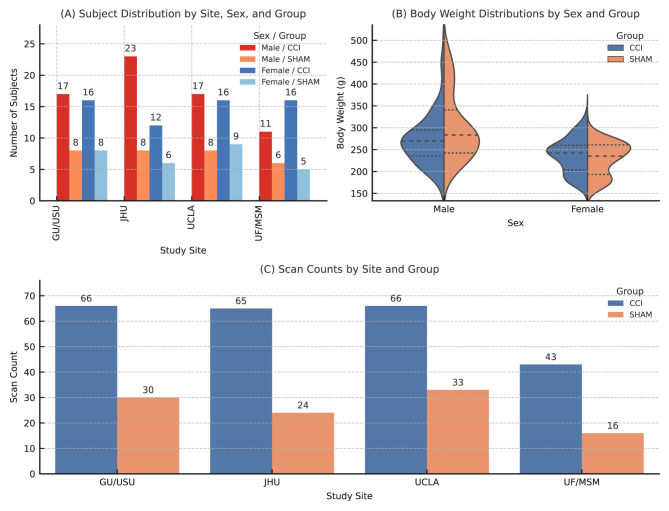
**(A)** Stacked bar plot of subject counts per study site, stratified by biological sex and experimental group (CCI or SHAM). Each bar segment represents the number of unique animals enrolled per subgroup. **(B)** Violin plots showing body weight distributions by sex and group at the subject level. Quartiles and median values are overlaid for each subgroup. **(C)** Bar plot of total scan counts per site, grouped by experimental condition (CCI or SHAM). Sample sizes (N) in this panel reflect the number of diffusion scans, not individual animals. Each animal was intended to contribute two scans (at day 3 and day 30 post-injury), but some records are missing due to either technical scanner-related problems or to occasional deaths.

To characterize the dataset prior to analysis, we visualized subject- and scan-level composition across study sites, experimental groups, and biological sex (Figure 1). Panel A displays the number of individual animals per site, showing balanced distribution across sex and injury groups, with some site-specific variation. Panel B presents body weight distributions at the time of scan acquisition, stratified by sex and experimental group, revealing expected weight differences between male and female rats, as well as slightly higher variability among SHAM animals. Panel C summarizes the total number of valid diffusion scans per site and group. Each animal was planned to undergo two diffusion MRI scans—on day 3 and day 30 post-injury—but missing records led to uneven scan availability across groups. These visualizations provide essential context for interpreting subsequent analyses and for understanding potential confounders such as inter-site imbalance and data completeness.

**Figure 2:**
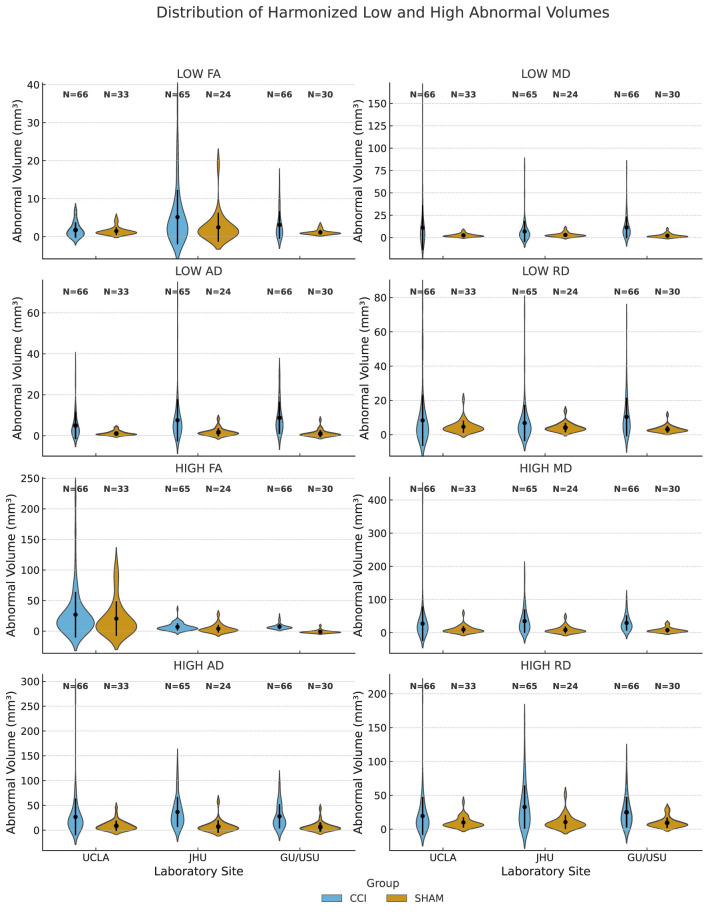
Distribution of voxel-wise harmonized low and high abnormal volumes by site and experimental group. Abnormal volumes (mm^3^) represent the number of brain voxels exceeding site-specific SHAM-derived *z*-score thresholds in harmonized DTI scalar maps. These maps include fractional anisotropy (FA), mean diffusivity (MD), axial diffusivity (AD), and radial diffusivity (RD). For each metric, abnormal low and high values were identified using voxel-wise *z*-scores computed relative to SHAM controls within each acquisition site and timepoint. Thresholds were based on corrected site-specific *z*-score cutoffs (typically |*z*| > 3.1). Results are grouped by acquisition site (UCLA, JHU, and GU/USU) and experimental condition (CCI or SHAM). Sample sizes (*N*) are indicated above each distribution.

To visualize the distribution of voxel-level scalar diffusion abnormalities across sites and experimental groups, we plotted harmonized abnormal volumes derived from FA, MD, AD, and RD maps using violin plots grouped by metric type (Figure 2). Each value corresponds to the brain-wide count of voxels in which the scalar metric significantly deviates from the local SHAM reference distribution, based on voxel-wise *z*-score maps registered to a common template space. Abnormal low and high volumes were defined using corrected *z*-thresholds determined per site and timepoint. The CCI group consistently displayed broader and more right-skewed distributions than SHAM animals, indicating a greater burden and variability of microstructural abnormalities. While harmonization reduced site-level differences, residual inter-site variability remained evident in both magnitude and spread. These distributions provide interpretable measures of scalar heterogeneity that may inform downstream statistical or machine learning analyses.

**Figure 3:**
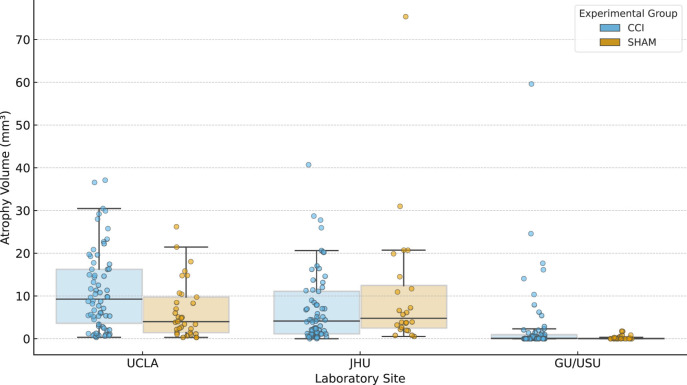
Range of brain atrophy volumes by experimental group and site. Atrophy volumes (mm^3^) are shown for three experimental sites (UCLA, JHU, and GU/USU), stratified by injury group (CCI, SHAM). Each value reflects the total volume of brain tissue exhibiting significant local contraction, computed from Jacobian determinant maps registered to a multi-site anatomical template. Individual scan-level data are displayed alongside boxplots, with sample sizes (*N*) indicated above each group. Atrophy volumes reflect the total volume of brain tissue exhibiting significant local contraction (Jacobian *z* < −3.1) and represent structural deformation following injury. These values may not align with microstructural abnormalities captured by diffusion metrics (see Figure 4) and thus reflect a distinct pathological dimension.

Figure 3 displays local tissue contraction data, referred to here as atrophy volume measurements for animals scanned at three of the four participating sites. Atrophy was defined as the total brain volume showing contraction at a Jacobian *z*-score threshold of less than −3.1. Measurements are grouped by acquisition site (UCLA, JHU, and GU/USU) and experimental group (CCI or SHAM). The data include both day 3 and day 30 post-injury scans and reflect deformation-based estimates of local tissue loss aligned to a harmonized multi-site template.

**Figure 4:**
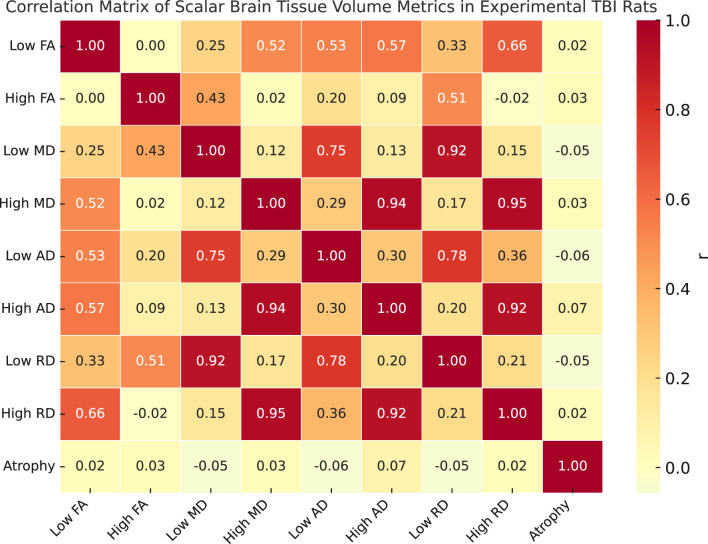
Correlation matrix of scalar brain tissue volume metrics (plotted on X and Y axis) in experimental TBI rats as a descriptive interpretation. Pairwise Pearson correlation coefficients are shown between nine scalar metrics derived from harmonized diffusion MRI data. The variables include low and high *z*-score volumes for fractional anisotropy (FA), mean diffusivity (MD), axial diffusivity (AD), and radial diffusivity (RD), along with total atrophy volume (Jacobian *z* < −3.1). Each entry in the matrix reflects the strength and direction of the linear relationship between two variables across the full dataset.

Each plotted value corresponds to a single scan session. The distributions summarize observed tissue contraction across animals within each group and site, without stratification by timepoint or sex. UF/MSM is not shown in this figure because it was determined to be a site outlier for diffusion data so that atrophy data were not required for further analysis and thus were not computed. While atrophy volumes quantify local structural contraction, they exhibit weak correlation with abnormal diffusion volumes across all scalar metrics (Figure 4).

Taken together, the atrophy and harmonized scalar abnormality distributions offer complementary views of injury-related structural and microstructural changes. Atrophy volumes capture macroscopic tissue loss typically associated with longer-term remodeling, while abnormal scalar volumes reflect acute or subacute microstructural disruptions (e.g., demyelination, edema, or axonal damage) identified through DTI-derived metrics (FA, MD, AD, and RD).

While both sets of measures show greater values in CCI animals compared to Shams, their distributions vary in shape and scale across sites and metrics, suggesting they capture distinct but complementary aspects of injury pathology.

Figure 4 presents a correlation matrix of harmonized scalar volume metrics and atrophy measurements computed from diffusion-weighted MRI scans. The scalar variables include eight harmonized tissue volume metrics derived from *z*-score thresholding of DTI scalars (FA, MD, AD, and RD), categorized into high and low *z*-score components. The atrophy variable represents total brain tissue volume showing significant contraction (Jacobian *z* < −3.1) based on nonlinear registration fields. The matrix displays Pearson correlation coefficients across all scans in the dataset. Variables were harmonized using NeuroCombat prior to correlation analysis. Strong positive correlations are observed between high-value diffusion metrics, particularly among MD and RD measures, as well as between low-value scalar pairs. Atrophy shows low to moderate correlation with scalar metrics, reflecting its complementary structural sensitivity.

## 5 Discussion

### 5.1 The value of the dataset

This dataset provides a high-quality, harmonized resource for investigating structural brain changes following traumatic brain injury (TBI) in a preclinical setting. Generated across four independent institutions using a standardized controlled cortical impact (CCI) model in adult Sprague Dawley rats, the dataset captures both acute and subacute injury phases through diffusion MRI and deformation-based anatomical metrics. By including both male and female subjects, two post-injury timepoints (3 and 30 days), and sham controls, it enables detailed analysis of injury severity, recovery dynamics, and sex-specific responses to brain trauma. One of the most significant contributions of the dataset is its rigorous cross-site harmonization. The use of NeuroCombat and voxel-level registration to a multi-site deformation template provides a rare level of standardization for preclinical neuroimaging data. This facilitates reproducibility and enables comparison of results across labs, which has historically been a challenge in animal model studies.

The dataset's scalar features—including FA, MD, AD, and RD—are enriched with corrected *z*-score–based injury volumes and atrophy estimates derived from Jacobian determinant fields. These features are widely recognized as relevant biomarkers of microstructural damage, white matter integrity, and neurodegeneration, supporting use in injury stratification, machine learning model development, and method benchmarking. In particular, the inclusion of harmonized and raw data allows researchers to evaluate the performance and impact of harmonization pipelines themselves. The structured metadata and integration of semantic provenance using PROV-O further enhance its value for data-driven research, enabling detailed reproducibility, interoperability, and machine-actionable traceability of every derived variable.

### 5.2 The limitations of the dataset

While the dataset provides a rich foundation for preclinical neurotrauma research, several limitations must be acknowledged. First, although the study includes multiple imaging sites, it is restricted to a single species (Sprague Dawley rats), a single injury model (CCI), and two discrete timepoints. This may limit its applicability to other experimental paradigms, injury mechanisms, or species. Second, despite the harmonization protocols in place, some site-specific differences in imaging hardware (e.g., magnetic field strength, gradient strength, coil configurations) may not be fully corrected by statistical adjustment, especially in small sample subgroups. Third, missingness is non-negligible in some voxel-harmonized scalar volume fields (~18%) due to failures in template registration or quality control exclusion. Although sufficient data remain for robust analysis, this may influence machine learning performance in downstream applications. Additionally, the dataset does not currently follow the Brain Imaging Data Structure (BIDS) specification, which would improve compatibility with clinical neuroinformatics pipelines. The effect of group size differences between sham and injured may contribute differences in the harmonization process but were not evaluated. Finally, while diffusion tensor imaging (DTI) metrics are informative, no complementary imaging modalities (e.g., T2-weighted, functional MRI) or histological validations are included, limiting multimodal interpretation.

### 5.3 Opportunities for future research

This dataset opens several avenues for advanced research in neurotrauma, neuroimaging harmonization, and AI-based biomarker discovery. The presence of harmonized and unharmonized scalar features makes it well-suited for evaluating the effect of preprocessing choices on downstream analyses, including classifier performance, feature stability, and cross-site generalization. Researchers can also explore temporal changes in scalar volumes to model recovery trajectories or identify early biomarkers predictive of later anatomical atrophy. Furthermore, the dataset could support training and benchmarking of neural networks and other AI systems, with voxel-level volumes offering a bridge between classical imaging metrics and modern deep learning feature spaces.

Future extensions of the dataset could include additional timepoints to capture chronic injury effects, integration of histological endpoints to validate imaging biomarkers, and adoption of BIDS-compliant structure to improve standardization. Combining this dataset with other open preclinical or clinical TBI datasets would allow for transfer learning experiments and cross-domain validation. Lastly, the strong alignment with FAIR² principles, including AI-readiness and responsible data practices, makes the dataset a compelling template for future multi-site neuroimaging initiatives aiming to meet emerging standards for transparency, provenance, and machine-actionable reproducibility.

## 6 Conclusion

Covering 343 diffusion-weighted MRI scans from 186 adult Sprague Dawley rats across four institutions, this dataset provides a harmonized, multi-site view of structural brain changes following controlled cortical impact (CCI). It captures both acute and subacute responses at 3 and 30 days post-injury, across CCI and sham groups, with representation of both sexes. The dataset includes unharmonized and harmonized scalar diffusion volumes (FA, MD, AD, RD), corrected *z*-score maps, and deformation-based atrophy measures, enabling analysis of microstructural disruption, recovery dynamics, and injury heterogeneity in a reproducible, preclinical setting.

A standardized processing pipeline—built on BrkRaw, FSL, MRtrix3, and ANTs—ensures spatial normalization, artifact correction, and consistent derivation of scalar metrics. Harmonization across imaging platforms is achieved through NeuroCombat and voxel-level registration to a shared anatomical template. Provenance is encoded using PROV-O to link scalar features directly to their generating methods, supporting auditability and machine-actionable traceability. The dataset adheres to FAIR² principles, offering findable, accessible, interoperable, and reusable content that is AI-ready and ethically transparent.

This resource is valuable for a wide range of stakeholders: neuroscientists studying TBI pathology and sex-specific responses, method developers testing harmonization algorithms, and data scientists building interpretable and fairness-aware models. It supports use cases ranging from group comparisons to unsupervised clustering, classifier development, and harmonization benchmarking. While its scope is limited to one species, two timepoints, and a single injury model, it serves as a rigorous reference for diffusion MRI–based biomarker development in preclinical neurotrauma.

Future directions include extending the dataset to chronic stages, integrating histological or behavioral data, and adopting BIDS formatting for greater interoperability. As a structured, transparent, and reproducible dataset, it lays the groundwork for responsible AI in preclinical imaging and contributes a critical benchmark for multi-site, open neuroimaging research.

## Data Availability

The dataset is FAIR^2^-certified and publicly available under an open license permitting unrestricted reuse with appropriate attribution. Access is provided through two coordinated components: an interactive FAIR^2^ Data Portal for visual exploration and quality assessment, and a downloadable FAIR^2^ Data Package containing harmonized data products, structured metadata, and detailed documentation. The original dataset is archived in the ODC-TBI repository under a persistent identifier (DOI: https://doi.org/10.34945/F5D60C), ensuring long-term preservation and linkage to the broader ODC-TBI data ecosystem. The FAIR^2^-compliant Data Package, which includes the interactive Data Portal, is published independently through the FAIR^2^ infrastructure with its own DOI (https://doi.org/10.71728/senscience.g7x2-a9k4) and is indexed in DataCite to support discoverability, reproducibility, and downstream integration.
